# Supplementary material for: Self-supervised pretraining in the wild imparts image acquisition robustness to medical image transformers: an application to lung cancer segmentation
Source: Proc Mach Learn Res. Author manuscript; Available in PMC 2025 Jan 17. (PMC11741178)
Supplement: 1 [file NIHMS2026189-supplement-1.pdf]

Appendix A. Additional analysis and results

A.1. Centered kernel alignment (CKA)

Feature similarities between pretrained/self-pretrained and fine tuned models were measured using centered kernel alignment (CKA), which computes a normalized similarity of two feature representations $\boldsymbol{X}$ and $\boldsymbol{Y}$ in terms of the Hilbert-Schmidt Independence Criterion (HSIC):

$$\mathrm{CKA}(\boldsymbol{K},\boldsymbol{L}) = \frac{\mathrm{HSIC}_0(\boldsymbol{K},\boldsymbol{L})}{\sqrt{\mathrm{HSIC}_0(\boldsymbol{K},\boldsymbol{K})\mathrm{HSIC}_0(\boldsymbol{L},\boldsymbol{L})}} \tag{1}$$

where $\boldsymbol{K}{=}\boldsymbol{X}\boldsymbol{X}^{\boldsymbol{T}}$ and $\boldsymbol{L}{=}\boldsymbol{Y}\boldsymbol{Y}^{\boldsymbol{T}}$ are the Gram matrices of feature $\boldsymbol{X}$ and $\boldsymbol{Y}$. CKA computation typically requires the feature activations of entire dataset to be stored in the memory, which is difficult to implement for transformers that have a large number of parameters. Hence, we implemented the minibatch CKA(Nguyen et al., 2020) by averaging HSIC scores over k minibatches as:

$$\mathrm{CKA_{minibatch}}(\boldsymbol{K},\boldsymbol{L}) = \frac{\frac{1}{\mathrm{k}}\sum_{\mathrm{i=1}}^{\mathrm{k}} \mathrm{HSIC}_1(\mathbf{X_i}\mathbf{X_i^T},\mathbf{Y_i}\mathbf{Y_i^T})}{\sqrt{\frac{1}{\mathrm{k}}\sum_{\mathrm{i=1}}^{\mathrm{k}} \mathrm{HSIC}_1(\mathbf{X_i}\mathbf{X_i^T},\mathbf{X_i}\mathbf{X_i^T})}\sqrt{\frac{1}{\mathrm{k}}\sum_{\mathrm{i=1}}^{\mathrm{k}} \mathrm{HSIC}_1(\mathbf{Y_i}\mathbf{Y_i^T},\mathbf{Y_i}\mathbf{Y_i^T})}} \tag{2}$$

An unbiased estimator of HSIC(Song et al., 2012) was computed to reduce dependency of CKA values on the batch size:

$$\mathrm{HSIC}_1(\boldsymbol{K},\boldsymbol{L}) = \frac{1}{n(n-3)}\left(\mathrm{tr}(\tilde{K}\tilde{L}) + \frac{\mathbf{1}^{\mathrm{T}}\tilde{K}\mathbf{1}\mathbf{1}^{\mathrm{T}}\tilde{L}\mathbf{1}}{(n-1)(n-2)} - \frac{2}{(n-1)}\mathbf{1}^{\mathrm{T}}\tilde{K}\tilde{L}\mathbf{1}\right) \tag{3}$$

A.2. Additional results

Table A.1. Robustness of tumor segmentation to different scan reconstructions. Significance tests compared wild-pretrained to self-pretrained and scratch trained models using the same network architecture.

Model	Training	Slice 2.5mm			Slice 5mm		
		Sharp	Smooth	p-value	Sharp	Smooth	p-value
CNN	Scratch	0.20±0.20	0.22±0.21	0.61	0.27±0.24	0.28±0.26	0.26
CNN	Self-pretraining	0.21±0.20	0.22±0.20	0.57	0.27±0.19	0.31±0.27	0.16
CNN	Wild-pretraining	0.23±0.21	0.24±0.23	0.68	0.30 ± 0.25	0.34±0.31	0.13
ViT	Scratch	0.47±0.34	0.54±0.32	0.08	0.49±0.34	0.50±0.30	0.64
ViT	Self-pretraining	0.64±0.18	0.56±0.25	0.019	0.58±0.26	0.51±0.23	0.14
ViT	Wild-pretraining	0.67±0.16	0.58±0.26	0.077	0.62±0.24	0.56±0.22	0.11
Swin	Scratch	0.52±0.32	0.36±0.36	0.37	0.57±0.48	0.47±0.34	0.12
Swin	Self-pretraining	0.58±0.27	0.54±0.31	0.13	0.52±0.28	0.49±0.30	0.058
Swin	Wild-pretraining	0.70±0.18	0.66±0.21	0.058	0.62±0.28	0.58±0.26	0.036

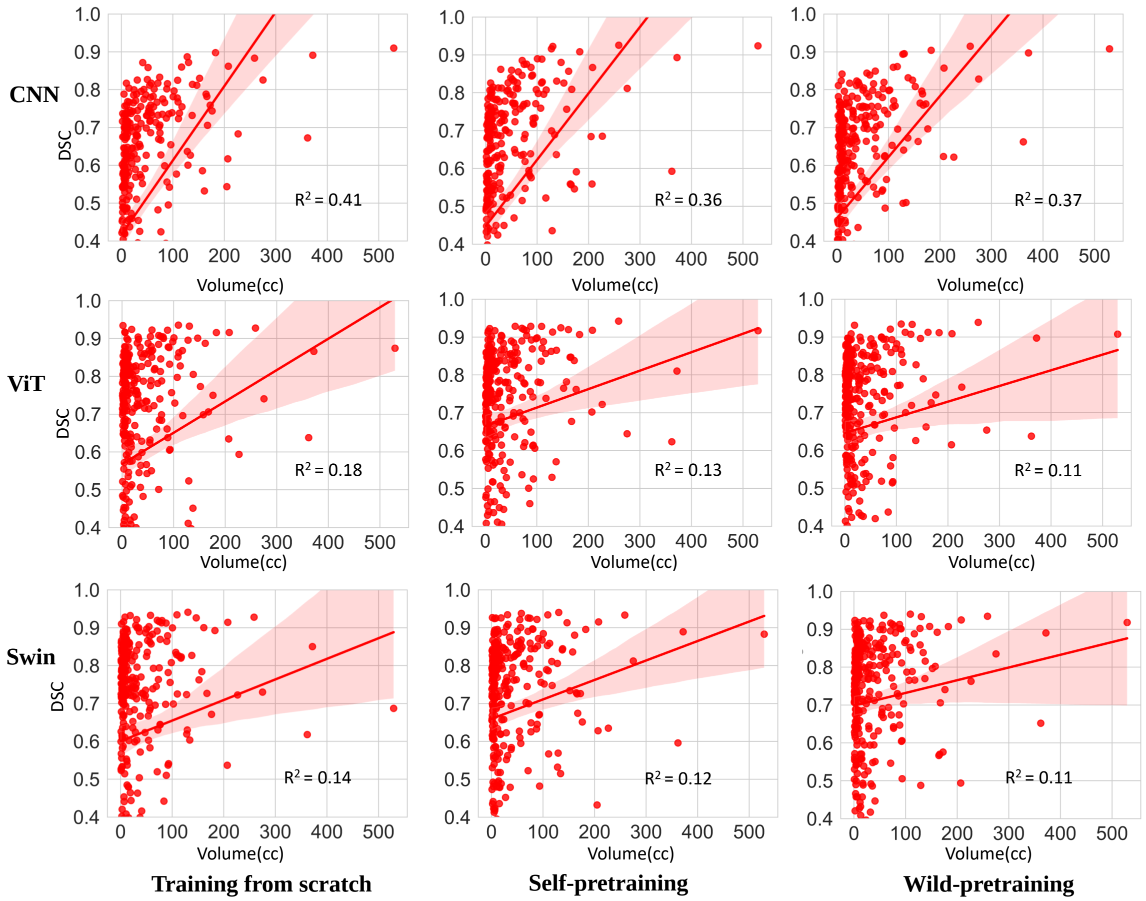

Figure A.1. The scatter plot of DSC versus tumor volume (cc) to assess dependency of accuracy on the tumor volume for the analyzed networks.

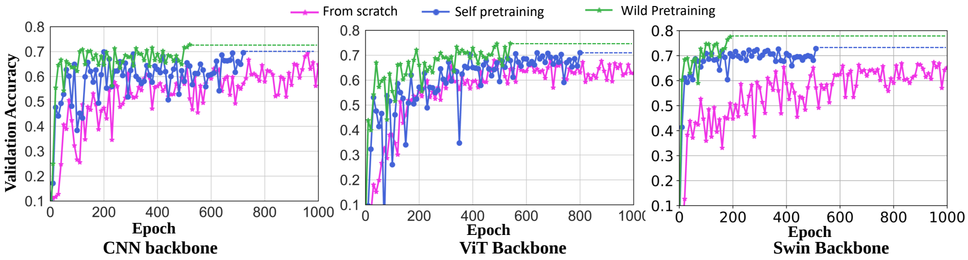

Figure A.2. Finetuning efficiency measured for self-pretrained and pretrained models using CNN, ViT and Swin backbone .
